# Symptomatic Protective Action of Glycyrrhizin (Licorice) in COVID-19 Infection?

**DOI:** 10.3389/fimmu.2020.01239

**Published:** 2020-05-28

**Authors:** Harald Murck

**Affiliations:** ^1^Department of Psychiatry and Psychotherapy, Philipps-University Marburg, Marburg, Germany; ^2^Murck-Neuroscience LLC, Westfield, NJ, United States

**Keywords:** Corona virus, COVID-19, glycyrrhizin, mineralocorticoid receptor, inflammation, toll like receptor 4 (TLR4), 11 beta hydroxysteroid dehydrogenase, angiotensin converrting enzyme

## Abstract

The role of the ACE2 enzyme in the COVID-19 infection is 2-fold, with opposing implications for the disease development. 1. The membrane bound angiotensin converting enzyme 2 (ACE2) serves as the entry point of COVID-19 2. Conversely, it supports an anti-inflammatory pathway. This led to the controversy of the impact of medications, which influence its expression. ACE2 is part of the wider renin-angiotensin-aldosterone system (RAAS) and is upregulated via compounds, which inhibits the classical ACE, thereby plasma aldosterone and aldosterone receptor (MR) activation. MR activation may therefore protect organs from binding the COVID-19 by reducing ACE2 expression. Glycyrrhizin (GL) is a frequent component in traditional Chinese medicines, which have been used to control COVID-19 infections. Its systemically active metabolite glycyrrhetinic acid (GA) inhibits 11beta hydroxysteroid dehydrogenase(11betaHSD2) and activates MR in organs, which express this enzyme, including the lungs. Does this affect the protective effect of ACE2? Importantly, GL has anti-inflammatory properties by itself via toll like receptor 4 (TLR4) antagonism and therefore compensates for the reduced protection of the downregulated ACE2. Finally, a direct effect of GL or GA to reduce virus transmission exists, which may involve reduced expression of type 2 transmembrane serine protease (TMPRSS2), which is required for virus uptake. Glycyrrhizin may reduce the severity of an infection with COVID-19 at the two stages of the COVID-19 induced disease process, 1. To block the number of entry points and 2. provide an ACE2 independent anti-inflammatory mechanism.

## Introduction

In the absence of primary prevention by immunization and a specific treatment for COVID-19 rationale treatment strategies may nevertheless be available. Besides therapies to affect virus replication directly [for overview see (1)], immunotherapies have been proposed to reduce the effects of the virus induced inflammation (2). Those include corticosteroid treatment, which are not recommended due to their immunosuppressive effects, which can lead to worse outcome in comparison to not treated subjects. More specific approaches target IL-6, TNFα, Janus kinase (JAK1/JAK2) inhibitors, and type 1 inteferons [ß1α and ß2α; see (2) for review]. Finally, the complement system has been considered as a target (3–5). These parameters may be of prognostic importance, as the ratio of IL6/interferon IFɤ appears to predict the severity of the disease (6).

Furthermore, the insight that the virus utilizes the membrane bound angiotensin converting enzyme 2 (ACE2) as an entry point opens up potential strategies to modify the activity of this system. It has been proposed that the use of angiotensin receptor blockers, which lead to an upregulated expression of ACE2, may be harmful (7). The alternative view of a potential beneficial effect of these compounds has also been expressed (8), based on the physiologically anti-inflammatory and protective effects of this enzyme. This controversy has recently been clearly outlined (9, 10). The challenge is to reduce the ACE2 as an entry point without making the inflammatory reaction worse, once an infection has occurred.

## ACE2 Reduction to Reduce Covid-19 Entry?

Following mechanistic findings reducing ACE2 expression would reduce the number of access points of the virus to the body during the primary infection and potentially the spread inside the body. Both should lead to a potentially milder clinical course. Cells, which are susceptible for the infection with SARS appear to be primarily type II pneumocytes, ileal absorptive enterocytes, and nasal goblet secretory cells (11). Therefore, it may be worthwhile to identify mechanism to reduce membrane ACE2 expression at these cells (having potential negative consequences in mind). To increase the plausibility of such an approach it would be useful to follow the reports of the successful use of traditional Chinese medicine (TCM) approaches. One of the most frequently used compounds of TCM contains an extract from glycyrrhiza glabra, i.e., the licorice plant (12) and interacts with the angiotensin-aldosterone system: One of its active constituents is glycyrrhizin (GL), which is metabolized in the gut of humans into the systemically active metabolite glycyrrhetinic acid (GA). GL and GA administration has a number of relevant effects: GA primarily inhibits an enzyme called 11-beta-hydroxysteroid dehydrogenase (11bHSD), both type 1 and 2 (13). Of relevance here appears type 2 (11bHSD2). Its inhibition allows cortisol to access mineralocorticoid receptors (MR) in aldosterone specific peripheral tissue, including the kidney, lung, nasal, and endothelial cells, in which it would be otherwise prevented to do so. This is by its activity to rapidly degrade cortisol intracellularly to allow aldosterone access to the receptor. In other words, an inhibition of this enzyme leads to an aldosterone like activation of MR via cortisol and may resemble the effects of high aldosterone levels in these organs. Of interest in this context is that high aldosterone levels lead to a downregulation of ACE2 in the kidney (14), a tissue, which expresses 11bHSD2 like the lung and nasal epithelial cells, i.e., main entry points for COVID-19, whereas MR antagonism has opposite effects in several tissues (15). This is in line with the observation that under certain circumstances aldosterone reducing compounds, like enalapril can lead to an increase of ACE2 expression (16, 17).

## Direct Antiviral Effect of GA or GL

Interestingly GL or its active metabolite GA expresses antiviral effects for the related SARS-corona virus (18, 19) in cell culture: Verum cells infected with patient plasma samples showed significantly reduced virus absorption and replication rate, when GL was co-administered (18); A similar effect has been described by Chen et al. in a Vero-E6 cell line, however, no effect was observed in an fRhK4 cell line (19). Importantly, GA, the systemically active compound after oral administration, was not studied, which makes these findings potentially relevant for local (inhaled) or intravenous administration. In a study with human respiratory tract cells GA, but not GL showed an effect on the infection rate with the human respiratory syncytial virus (20). These direct antiviral effects outside of MR point to an additional, but unknown mechanism. In this context it may be important to note that in addition to ACE2 the serine protease TMPRSS2 is required for the infection of a cell (21). The inhibition of this enzyme by a protease inhibitor as a therapeutic intervention has been proposed by the authors. TMPRSS2 has been involved in both corona and influenza virus infections (22). Interestingly, this expression of this enzyme is regulated by GA (23), which may account for the broader antiviral effects of GL (24). It is regulated by androgens (23), which may explain in part the gender differences in the clinical expression of COVID-19 infections.

## ACE2 Antiinflammation and Glycyrrhizin's Anti-Inflammatory Effects

The downstream consequences of reduced ACE2 expression are, as outline above, somewhat controversial (25). ACE2 activity is generally protective, including for lung tissue (26). It does so by suppressing the consequences of the activation of the receptor for endotoxin (LPS), i.e., the toll-like receptor 4 (TLR4) and as a consequence related inflammation in the lung (endotoxin storm) (27): ACE2 overexression inhibited the LPS induced inflammation in this study. Therefore, the reduced expression of ACE2 could be regarded as concerning. In this context a second property of glycyrrhizin becomes important, i.e., its immunmodulatory effect. The best knows of these is its antagonistic effect of TLR4 dependent mechanims. A TLR4 antagonistic effect of GA reduces inflammation in several tissues, including the lung (28). In addition, GL lead to a reduction of TLR4 expression in the heart and the lung in an LPS model of inflammation. This was accompanied by a significant reduction of cytokine release, i.e., the release of TNFα, IL6, and IL1ß (29). In accordance GL has protective effects in acute respiratory distress syndrome induced by the TLR4 activator LPS in mice (28). The anti-inflammatory potential within the lung was also demonstrated in a mouse model of Streptococcus aureus infection, where intraperitoneal administration of GL suppressed inflammatory markers broadly (30). These findings are in line with the activity of GL or GA to inhibit inflammatory pathways, via TLR4 (31–34). The mechanism of GL against lung and cardiac inflammation may in part be indirect by altering the ratio of myeloid derived suppressor cells (MDSCs) to CD11b+Gr1 myeloid cells (29). Overall, the action of GL to inhibit TLR4 activity may induce an anti-inflammatory activity downstream of the less active ACE2 (35). In addition, GA leads to a suppression of the classical, but not the alternative complement pathway (36). Finally an influence on interferone secretion has been described: glycyrrhiza extract leads to an increased secretion of interferon 1ß in upper and lower respiratory tract cells (20) similar to the effect of GL and GA in mice, as determines in serum samples (37); furthermore GL reduces death in mice infected with a lethal dose of influenza virus via an interferon ɤ and T-cell dependent way (38). These antiinflammatory mechanisms may also be of importance in the CNS (39, 40) and may therefore protect against neurological and psychiatric consequences of a COVID-19 infection.

**Figure 1:**
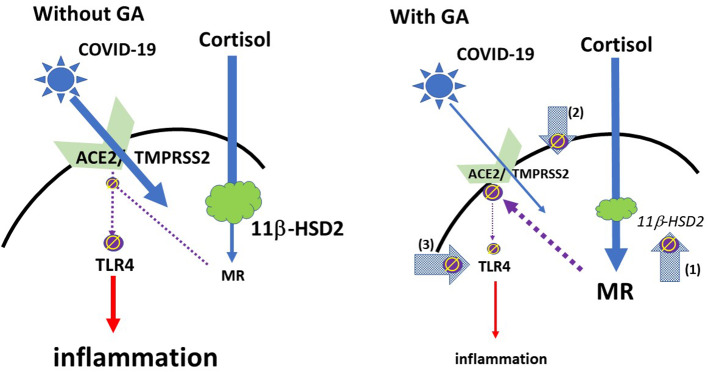
Schematic model of the effect of glycyrrhizin: COVID-19 access into cells is mediated via ACE2 with TMPRSS2 as a co-factor. The expression of ACE2 is regulated by mineralocorticoid receptors (MR): MR activation leads to a reduction of ACE2 expression; GA inhibits the 11βHSD2, which allows cortisol to activate MR, followed by ACE2-downregulation (arrow 1). TMPRSS2 sensitizes ACE2 for the update of the virus into the cell. GA leads to a reduced expression of TMPRSS2 and may therefore provide an additional mechanism to restrict the virus' access into the cell (arrow 2). ACE2 has an anti-inflammatory mechanism by the generation of angiotensin 1-7 and angiotensin 1-9. Via activation of MAS or angiotensin 2 receptors inflammatory pathways are suppressed. This also includes a reduced expression and/or activation of the membrane TLR4 receptor (left), i.e., the reduced ACE2 expression could be regarded as problematic (35). However, GA directly inhibits TLR4 independent of ACE2 activation (Arrow 3). (ϕ and interrupted lines symbolize inhibition; red continuous lines symbolize activation).

From a more practical perspective it may also be relevant that the coronavirus SARS Co-V, which has similarities to COVID-19, led frequently to arterial hypotension (25), which is not uncommon in inflammatory processes. This potentially critical symptom may also be overcome with glycyrrhizin, which leads to an increase in blood pressure (13). Please see Figure 1 for a schematic overview.

## Discussion

Many questions remain open. What is the role of soluble vs. membrane bound ACE2? May there be a role of soluble ACE2 to protect membrane occupancy? This has recently been proposed on the basis of findings in cell culture experiments (41). This may, however, somewhat contradict the observation that subjects with cardiac failure, who should be regarded as high risk, show high levels of soluble ACE2 (42). What is the difference in different organs with or without 11bHSD2 expression? What is the role of the concomitant counterregulatory reduction of plasma aldosterone with the administration of a 11betaHSD2 inhibitor? These questions can be answered in appropriate clinical trials. The determination of the end-product of the ACE2 enzyme, i.e., angiotensin 1-7 as well as potential clinical consequences on blood pressure may be helpful to clarify some of these issues.

Importantly, glycyrrhizin has an overall well-tolerated. It has an FDA statement of GRAS (generally regarded as safe) (13). In particular, a dose up to 100 mg/day used chronically is safe and does not lead to changes, which have been observed with chronic use in higher doses. The expected unwanted effects of high doses, including hypertension and hypokalemia, should however be monitored. In the context of SARS an oral dose of up to 300 mg has been recommended for oral administration and of approximately 240 mg for an intravenous administration (19). However, Chen et al. state that this dose for the i.v. administration may be too low, taking the EC50 of the effect on virus replication into account. It has to be stated that this direct effect is only one of three relevant mechanism to target the COVID-19 related disease process. Two open label clinical trials are registered on the WHO clinical trial registration website, a randomized open label trial (ChiCTR2000029768) and a case series (ChiCTR2000030490). For the trial a dose of 300 mg glycyrrhizin orally/day is used, the dose for the other investigation was not reported. For the choice of the administration path it is important to consider that for a GA induced action the oral administration of GL is crucial, as GL is not metabolized to GA systemically. However, for a localized effect of GL an intravenous or inhaled administration may be required, which should be combined with an oral administration. For the use of a potential primary prevention a pragmatic dose selection of 150 mg/day orally may be considered for further studies, as this dose affects the activity of the MR.

## Conclusion

Glycyrrhizin is a widely available and overall safe compound. It may be capable of reducing the expression of ACE2 in the lung and despite that reduce lung inflammation. It should be worth a consideration to study this compound for a type of primary prevention, which does not necessarily lower the risk of becoming infected, but potentially the severity of the disease, and in reducing already existing symptomatology. This could help reduce the number of critically ill patients, which currently overwhelm the healthcare system.

## Author Contributions

HM drafted the manuscript and the final version.

## Conflict of Interest

HM holds a patent for the use of glycyrrhizin in therapy refractory depression. The author declares that the research was conducted in the absence of any commercial or financial relationships that could be construed as a potential conflict of interest.
